# Prevalence of Dry Eye Disease in Patients With Diabetes Mellitus: A Systematic Review

**DOI:** 10.7759/cureus.109762

**Published:** 2026-05-27

**Authors:** Ashley JY. Lim, Jessica Vasantharasan, Akshaj Jonnalagadda, Victoria C Kaplan, Marya A Alfurayj

**Affiliations:** 1 School of Medicine, University College Dublin, Dublin, IRL

**Keywords:** diabetes mellitus, dry eye disease, ocular surface, prevalence, screening

## Abstract

Dry eye disease (DED) is a multifactorial disorder of the ocular surface increasingly recognised as a complication of diabetes mellitus (DM). Prevalence estimates in DM patients vary widely due to differences in diagnostic methods and populations. This systematic review aimed to synthesise contemporary evidence on DED prevalence in DM patients and explore factors influencing variability, particularly diagnostic approaches. A systematic search was conducted in four databases (PubMed, Embase, Scopus, Web of Science) for studies published between January 2015 and June 2025. Eligible studies included those reporting the prevalence of DED in patients with type 1 or type 2 DM, using clinical assessments or validated diagnostic methods. Only peer-reviewed, full-text studies published in English were included. Following an initial screening of 2053 records, 18 studies met the inclusion criteria for qualitative synthesis. Risk of bias was assessed using the Newcastle-Ottawa Scale. Prevalence ranged from 6.6% to 72.3%. Rates were lowest in large administrative studies using the International Classification of Diseases (ICD) codes (6.6-14.4%), intermediate in questionnaire-only assessments (34.8-51.7%), and highest in multimodal clinical evaluations combining symptoms with objective tests (17.5-72.3%). Where non-diabetic controls were included, DED prevalence was consistently higher in DM patients. DED is highly prevalent in DM patients, with estimates strongly influenced by diagnostic sensitivity. This suggests underdiagnosis where administrative data or symptoms alone are used. Routine screening with sensitive multimodal tools is recommended in DM care. Future research should adopt standardised diagnostics and broader geographic representation to enable quantitative synthesis.

## Introduction and background

Dry eye disease (DED) is a multifactorial ocular surface condition characterised by tear film instability, hyperosmolarity, ocular surface inflammation, and neurosensory abnormalities. Patients typically present with symptoms including dryness, burning, foreign body sensation, and fluctuating vision. Population-level prevalence estimates vary widely, from 5% to 50%, reflecting genuine epidemiological differences across age groups and regions, but also the absence of a universally adopted diagnostic standard [1].

Diabetes mellitus (DM) is one of the most prevalent chronic diseases worldwide, affecting over 500 million adults and projected to rise substantially over the coming decades. While diabetic retinopathy, nephropathy, and neuropathy are well-established microvascular complications, the ocular surface has received comparatively less attention in both research and clinical practice. Chronic hyperglycaemia disrupts corneal nerve integrity, reduces goblet cell density, impairs lacrimal gland function, and alters the composition of the tear film, all of which predispose patients with DM to DED [2]. Diabetic peripheral neuropathy affecting corneal sensory fibres further compounds this risk by reducing blink reflex sensitivity and impairing the afferent limb of the lacrimal reflex arc.

Despite a growing body of literature on DED in DM, reported prevalence estimates remain highly inconsistent. Differences in diagnostic criteria, the use of symptom questionnaires versus objective clinical tests, study population characteristics, diabetes type and duration, and glycaemic control all contribute to this variability [3]. Some studies report markedly elevated DED prevalence in patients with DM compared to healthy controls, while others report no significant difference, making it difficult to draw firm conclusions about the true burden of disease. This inconsistency has practical consequences: without reliable prevalence data, the case for systematic screening in diabetes care pathways remains underdeveloped.

This systematic review aimed to synthesise contemporary prevalence estimates of DED among patients with DM and to examine how diagnostic approach, study design, and population characteristics influence reported prevalence.

## Review

Methods

This systematic review (International Prospective Register of Systematic Reviews (PROSPERO) ID: CRD420251085551) was conducted following the Preferred Reporting Items for Systematic Reviews and Meta-Analyses (PRISMA) guidelines [4]. A search was conducted across four databases: PubMed, Embase, Scopus, and Web of Science. The search covered studies published between January 2015 and June 2025 and included the following key terms: "dry eye", "dry eye disease", "ocular surface disease", "keratoconjunctivitis sicca", "diabetes", "diabetes mellitus", "type 1 diabetes", "type 2 diabetes", "prevalence", "epidemiology", "incidence", "frequency", and "occurrence". Full database-specific search strategies, including Boolean operators and Medical Subject Headings (MeSH)/Emtree terms for each database, are provided in the Appendices section. 

Studies were eligible for inclusion if they met all of the following criteria: (1) full-text articles published in English; (2) involved human participants with a confirmed diagnosis of type 1 DM (T1DM) or type 2 DM (T2DM); (3) reported the prevalence of DED using clinical assessments, validated diagnostic instruments, or administrative coding systems; (4) included at least 100 participants with DM to ensure the adequate precision of prevalence estimates; and (5) were published within the specified date range. DED diagnosis was defined according to each study's own criteria, encompassing symptom-based approaches using validated questionnaires, objective clinical signs, or administrative International Classification of Diseases (ICD) codes. Adherence to a single universal framework such as the Tear Film and Ocular Surface Society Dry Eye Workshop II (TFOS DEWS II) definition was not required for inclusion, reflecting the real-world variability in diagnostic practice across settings and time periods.

Studies were excluded if they were non-original research (e.g., reviews, case reports), animal studies, and conference abstracts only or if they focused exclusively on non-diabetic populations or ocular conditions unrelated to DED.

**Figure 1:**
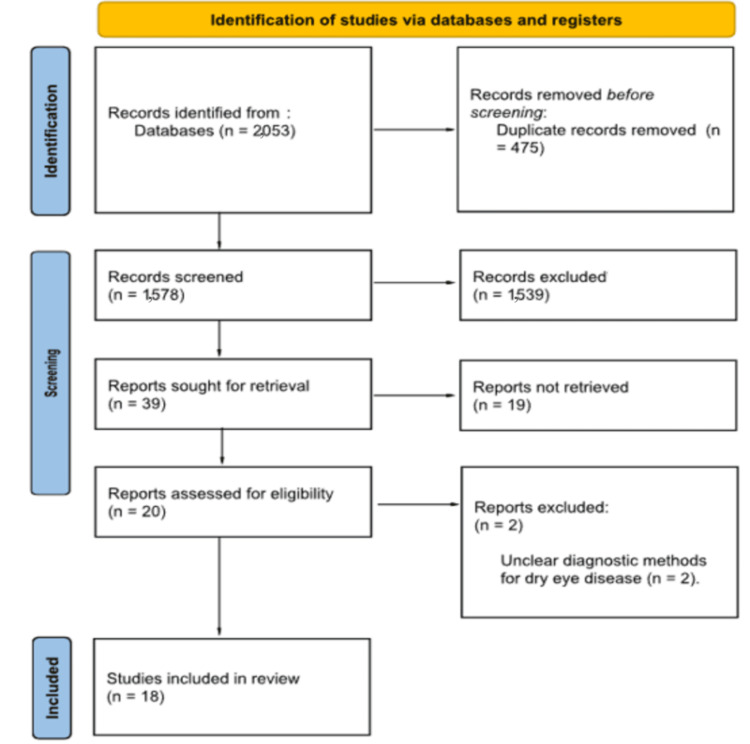
PRISMA flowchart. PRISMA: Preferred Reporting Items for Systematic Reviews and Meta-Analyses

A total of 2,053 records were retrieved from the initial database search. After the removal of duplicate entries, 1,578 records were screened at the title and abstract level by two independent reviewers. Full-text review was then performed for records meeting preliminary eligibility criteria. Of the 39 records selected for full-text retrieval, 20 full texts were successfully retrieved and assessed for eligibility. The remaining 19 records could not be retrieved as they were conference abstracts or proceedings without an associated full-text publication. Their exclusion is unlikely to introduce systematic bias, as they were not concentrated within any single diagnostic subgroup or geographic region. After the screening, 18 studies remained for inclusion in the final review. Discrepancies between reviewers at any stage were resolved through discussion and, where necessary, adjudication by a third reviewer. The full screening and selection process is presented in the PRISMA flowchart (Figure 1).

Results

Study Characteristics

The 18 included studies were conducted across multiple geographical regions, with the majority originating from Asia and sub-Saharan Africa. Sample sizes ranged from 100 to 152,520 participants. Participant age ranged broadly, with mean or median ages typically falling between the fourth and sixth decades. Gender distribution was generally balanced across studies, with some reporting a slight female predominance. Most studies focused exclusively on T2DM, and no study exclusively examined T1DM. Studies involving mixed populations reported T1DM and T2DM prevalence together without consistent stratification.

Diagnostic approaches varied considerably. The most frequently used assessments were the Ocular Surface Disease Index (OSDI) questionnaire, Tear Break-Up Time (TBUT), and Schirmer's test, often used in combination with ocular surface staining using fluorescein, Rose Bengal, or lissamine green and slit lamp biomicroscopy. Additional tools included the Standardized Patient Evaluation of Eye Dryness (SPEED) questionnaire, Dry Eye Questionnaire (DEQ-5), McMonnies questionnaire, tear meniscus height measurement, and meibomian gland assessment. Two large-scale studies relied on administrative ICD codes for case identification. This diversity in diagnostic approaches directly influenced reported prevalence and was the primary basis for subgroup stratification in the analysis.

Risk of bias was assessed using the Newcastle-Ottawa Scale (NOS) for cohort and cross-sectional studies [5,6] and the Joanna Briggs Institute (JBI) Critical Appraisal Checklist for case series studies [7]. Each study was independently assessed by two reviewers using the relevant instrument. Discrepancies in individual domain scores were resolved by discussion; where consensus was not reached, a third reviewer adjudicated. Inter-rater agreement was high (Cohen's kappa = 0.81 prior to resolution), indicating strong consistency in quality assessment. Domains assessed included selection, comparability, and outcome or exposure assessment. Scores were categorised as low (0-3), moderate (4-6), or high quality (7-9). The majority of the included studies demonstrated moderate to high quality. The study characteristics and quality assessments are summarised in Table 1 and Table 2.

**Table 1:** Summary of the included studies. T1DM: type 1 diabetes mellitus; T2DM: type 2 diabetes mellitus; DED: dry eye disease; DR: diabetic retinopathy; IQR: interquartile range; ICD: International Classification of Diseases; TBUT: Tear Break-Up Time; OSDI: Ocular Surface Disease Index; DEQS: Dry Eye-Related Quality-of-Life Score; OCT: optical coherence tomography; MOGS: Modified Oxford Grading Scheme

Study	Study type	Country	Age (years)	Gender (male:female)	Diagnostic tool(s) for DED
Zou et al., 2018 [8]	Community-based cross-sectional study	China	Mean: 68.91 ± 8.86; range: 31-95	507:853	Symptom questionnaire, TBUT, Schirmer I test, fluorescein and lissamine green staining, corneal sensitivity test
Ward et al., 2019 [9]	Retrospective cross-sectional study	United States	Age groups: 18-34, 35-54, 55-64, ≥65	35,004:46,476	ICD-9 (375.15, 370.33), ICD-10 (H04.12, H16.221) codes
Waris et al., 2019 [10]	Hospital-based prevalence study	India	Not reported	Not reported	Standard Dry Eye Questionnaire, TBUT, Schirmer's test, fluorescein staining, Rose Bengal staining, slit lamp biomicroscopy
Yazdani-Ibn-Taz et al., 2019 [11]	Cross-sectional study	United Kingdom	T1DM: 47 ± 17; T2DM: 61 ± 13	87:65	OSDI questionnaire, DEQS
Dutta et al., 2021 [12]	Clinical observational study	India	Mean: 54.52; range: 35-70	51:49	OSDI questionnaire, TBUT, Schirmer's test, fluorescein staining, Rose Bengal staining, conjunctival impression cytology
Abu et al., 2022 [13]	Hospital-based cross-sectional study	Ghana	Mean: 59.85 ± 10.04; range: 35-85	71:240	OSDI questionnaire, Schirmer's test 1, TBUT, ocular surface staining (Oxford Grading Scale), blink rates
Almohammed et al., 2022 [14]	Retrospective cross-sectional survey-based study	Saudi Arabia	≥20	182:207	OSDI questionnaire
Pan et al., 2022 [15]	Retrospective population-based cohort study	Taiwan	DED group: 63.7 ± 12.4; non-DED group: 63.7 ± 12.4	87,142:65,378	ICD codes: ICD-9-CM, ICD-10-CM
Bekele et al., 2023 [16]	Hospital-based cross-sectional study	Ethiopia	Median: 56; IQR: 47-65; range: ≥18	260:228	OSDI questionnaire
Bhandary et al., 2023 [17]	Prospective study	India	Range: >30	64:56	OSDI questionnaire, Schirmer's test, TBUT, fluorescein staining pattern, slit lamp biomicroscopy, indirect ophthalmoscopy
Giriswamy, 2023 [18]	Hospital-based study	India	Diabetic patients without retinopathy: 53.50 ± 10.77; diabetic patients with retinopathy: 56.99 ± 8.40	Without DR: 58:42; with DR: 55:45	OSDI questionnaire, TBUT, ocular surface staining with fluorescein, Schirmer's test, slit lamp biomicroscopy
Brar et al., 2024 [19]	Hospital-based cross-sectional observational study	India	Range: 40-89	123:77	Rose Bengal staining, Schirmer's I and II tests, TBUT
Tran Tat et al., 2024 [20]	Cross-sectional study	Vietnam	64.19 ± 10.21	163:175	OSDI questionnaire, TBUT
Zekaj et al., 2024 [21]	Case-series study	Kosovo	62.4 ± 9.7	196:204	OSDI questionnaire, Schirmer's test, TBUT, OCT
Ansar et al., 2025 [22]	Cross-sectional observational study	India	Mean: 56.02 ± 9.29; range: 40-75	115:88	OSDI questionnaire, Schirmer's I test, TBUT, MOGS
Pandey et al., 2025 [23]	Cross-sectional study	India	With DR: 52.32 ± 9.36; without DR: 51.30 ± 9.02	With DR: 32:18; without DR: 33:17	OSDI questionnaire, Schirmer's test, McMonnies Dry Eye questionnaire
Patel, 2025 [24]	Prospective observational study	India	Range: 30-80	50:50	Schirmer's test, TBUT, fluorescein staining
Shumye et al., 2025 [25]	Multicentre institution-based cross-sectional study	Ethiopia	Median: 53; IQR: 37-62	619:515	OSDI questionnaire

**Table 2:** Quality assessment of the included studies. D1: Were there clear criteria for inclusion in the case series? D2: Was the condition measured in a standard, reliable way for all participants included in the case series? D3: Were valid methods used for the identification of the condition for all participants included in the case series? D4: Did the case series have consecutive inclusion of participants? D5: Did the case series have complete inclusion of participants? D6: Was there clear reporting of the demographics of the participants in the study? D7: Was there clear reporting of clinical information of the participants? D8: Were the outcomes or follow-up results of cases clearly reported? D9: Was there clear reporting of the presenting site(s)/clinic(s) demographic information? D10: Was statistical analysis appropriate?

Cross-sectional studies											
Study	Selection (max 5 stars)			Comparability (max 2 stars)			Outcome/exposure (max 3 stars)			Total score	
Zou et al., 2018 [8]	****			**			**			8	
Ward et al., 2019 [9]	***			**			***			8	
Waris et al., 2019 [10]	**			**			**			6	
Yazdani-Ibn-Taz et al., 2019 [11]	**			**			**			6	
Dutta et al., 2021 [12]	***			*			**			6	
Abu et al., 2022 [13]	****			**			***			9	
Almohammed et al., 2022 [14]	****			**			**			8	
Bekele et al., 2023 [16]	****			**			**			8	
Giriswamy, 2023 [18]	***			**			**			7	
Brar et al., 2024 [19]	**			**			**			6	
Tran Tat et al., 2024 [20]	**			**			**			6	
Ansar et al., 2025 [22]	***			**			**			7	
Pandey et al., 2025 [23]	***			**			**			7	
Shumye et al., 2025 [25]	****			**			**			8	
Cohort studies											
Study	Selection (max 4 stars)			Comparability (max 2 stars)			Outcome/exposure (max 3 stars)			Total score	
Pan et al., 2022 [15]	****			**			***			9	
Bhandary et al., 2023 [17]	***			**			***			8	
Patel, 2025 [24]	**			**			***			7	
Case series											
Study	D1	D2	D3	D4	D5	D6	D7	D8	D9	D10	Overall appraisal
Zekaj et al., 2024 [21]	Yes	Yes	Yes	Yes	Yes	Yes	Yes	Yes	Yes	Yes	Include

Prevalence of DED

Reported prevalence ranged from 6.6% to 72.3% across all included studies. Most studies focused on T2DM populations, within which prevalence varied from 6.6% in a large retrospective administrative study to 72.3% in a smaller cross-sectional clinical study [12,14]. Studies involving mixed or unspecified DM populations reported prevalence between 14.4% and 51.7%. Across all diagnostic approaches, most studies reported DED prevalence above 30%, underscoring the high burden of ocular surface disease in this population. A summary of DED prevalence across all the studies is presented in Table 3.

**Table 3:** Prevalence of DED across the included studies. ᵃGiriswamy [18] compared diabetic patients with diabetic retinopathy (68%) vs without diabetic retinopathy (32%). DED: dry eye disease; DM: diabetes mellitus; ICD: International Classification of Diseases

Study	Total DM patients	DM type	Prevalence of DED in DM patients (%)	Prevalence of DED in the control group (%)
Multimodal clinical tests				
Zou et al., 2018 [8]	1,360	2	17.5	5.9
Waris et al., 2019 [10]	100	2	43	No control group
Yazdani-Ibn-Taz et al., 2019 [11]	110	1 and 2	44	29
Dutta et al., 2021 [12]	100	2	48.3	No control group
Abu et al., 2022 [13]	311	2	72.3	No control group
Bhandary et al., 2023 [17]	120	Not specified	50.9	No control group
Giriswamy, 2023 [18]	100	2	55	See noteᵃ
Brar et al., 2024 [19]	200	2	31.5	No control group
Tran Tat et al., 2024 [20]	169	2	37.3	No control group
Zekaj et al., 2024 [21]	400	2	33	No control group
Ansar et al., 2025 [22]	203	2	60.1	No control group
Pandey et al., 2025 [23]	100	2	30	No control group
Patel, 2025 [24]	100	2	71	No control group
Questionnaires				
Almohammed et al., 2022 [14]	389	1 and 2	51.7	No control group
Bekele et al., 2023 [16]	488	1 and 2	34.8	No control group
Shumye et al., 2025 [25]	1,134	1 and 2	40.4	No control group
ICD codes/administrative database codes				
Ward et al., 2019 [9]	18,361	1 and 2	14.4	10.1
Pan et al., 2022 [15]	152,520	2	6.6	No control group

**Figure 2:**
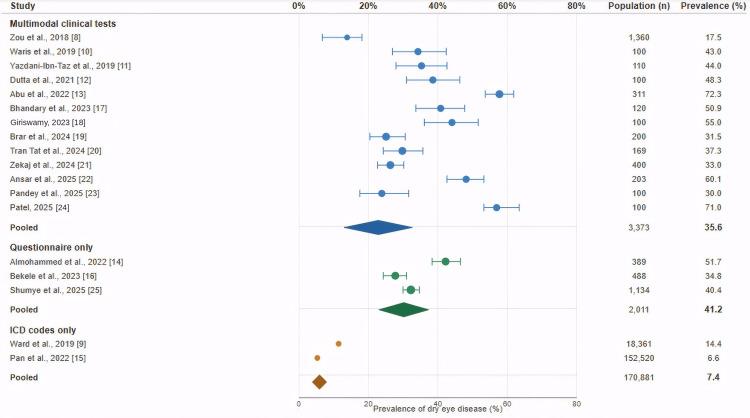
Forest plot displaying subgroup-stratified prevalence estimates for DED in patients with DM. DED: dry eye disease; DM: diabetes mellitus; ICD: International Classification of Diseases

Given the substantial heterogeneity in diagnostic methods and study designs, a single overall pooled prevalence estimate was not calculated. Studies were stratified by diagnostic approach: ICD/administrative codes, validated symptom questionnaires (OSDI, SPEED, DEQ-5, McMonnies), and multimodal clinical tests (TBUT, Schirmer's test, ocular surface staining, or slit lamp biomicroscopy). Subgroup pooled prevalence estimates were calculated using a random-effects model (DerSimonian-Laird method) with Freeman-Tukey double arcsine transformation applied prior to pooling. Heterogeneity within each subgroup was quantified using the I² statistic. Confidence intervals were estimated at the 95% level. Pooled estimates with 95% confidence intervals are displayed in Figure 2 alongside individual study point estimates.

Subgroup pooled prevalence estimates were 35.6% for multimodal clinical tests (13 studies; n = 3,373), 41.2% for questionnaire-only studies (three studies; n = 2,011), and 7.4% for ICD/administrative code studies (two studies; n = 170,881). Heterogeneity was high within all three subgroups, reflecting the diversity of populations, geographic settings, and diagnostic instruments across the included studies.

Discussion 

This systematic review synthesised 18 studies enrolling over 176,000 participants to estimate the prevalence of DED in patients with DM. The findings confirm an elevated burden of DED in diabetic populations, with prevalence ranging from 6.6% to 72.3% across the included studies. Diagnostic heterogeneity was the primary driver of this variability, with lower rates consistently observed in studies relying on administrative coding and higher rates in those using multimodal clinical assessments. DED emerged as a frequent and clinically significant comorbidity of DM, particularly T2DM. These findings extend prior systematic reviews by incorporating more recent, larger cohorts and by explicitly quantifying the diagnostic gradient in reported prevalence.

Interpretation of Findings

The association between DM and increased DED risk is well-established in the epidemiological literature [1,26,27], and the findings of this review are consistent with that body of evidence. A prior systematic review and meta-analysis reported a pooled odds ratio of approximately 2.30 and a relative risk of 1.69 for DED in individuals with DM compared to those without [27]. This review complements that evidence by providing absolute prevalence estimates drawn from larger and more contemporary cohorts, offering a clearer picture of the current clinical burden at a population level.

Diagnostic method was the dominant source of heterogeneity across studies, a pattern well-recognised in DED epidemiology more broadly [1,28]. Studies using multimodal objective assessments, combining the OSDI questionnaire with TBUT, Schirmer's test, and ocular surface staining, reported the highest prevalence estimates, as these tools detect both symptomatic and subclinical disease. Administrative ICD-based studies produced the lowest estimates, capturing only cases formally diagnosed and coded in clinical encounters, which likely reflects severe or treatment-seeking cases rather than true population prevalence [29]. Questionnaire-only approaches produced intermediate estimates, relying on patient-reported symptoms that are subject to recall bias, variation in symptom awareness, and differing score thresholds for case classification. This diagnostic sensitivity gradient has direct implications for clinical practice: patients assessed only through symptom questionnaires or administrative records are at risk of having DED missed entirely, particularly in resource-limited settings where objective testing is less accessible.

The pathophysiological basis for the elevated DED prevalence in DM is multifactorial [30]. Chronic hyperglycaemia damages corneal sensory nerves, reducing blink reflex sensitivity and impairing the afferent limb of the lacrimal reflex arc. Lacrimal gland dysfunction reduces aqueous tear production, while meibomian gland alterations compromise the lipid layer of the tear film, accelerating evaporative loss. Concurrently, oxidative stress and advanced glycation end products promote ocular surface inflammation, further destabilising the tear film [2]. Although inconsistent confounder reporting across the included studies prevented formal subgroup analysis, several studies identified associations between DED severity and poorer glycaemic control as measured by hemoglobin A1c (HbA1c), longer DM duration, and concurrent diabetic retinopathy [31-33]. Beyond the diagnostic method, other factors contribute to the variability in reported prevalence. Age is an independent risk factor for DED and likely explains some of the higher rates observed in older hospital-based cohorts [1]. Longer DM duration and poorer glycaemic control, as reflected by elevated HbA1c, were associated with higher DED severity in several studies, supporting a neuropathic and inflammatory mechanism. Geographic and climatic differences may also play a role: studies from sub-Saharan Africa report a range of prevalence figures that partially reflect differences in humidity, air quality, and healthcare-seeking behaviour, in addition to diagnostic approach. These factors were inconsistently reported across the included studies and precluded formal subgroup analysis, but they represent important sources of unexplained heterogeneity that future research should address through standardised reporting. These associations position DED as a microvascular and neuropathic complication of DM, mechanistically comparable to retinopathy and nephropathy, and reinforce the argument for its inclusion in routine diabetic eye assessments [34].

Strengths and Limitations

This review has several methodological strengths. The minimum sample size threshold of 100 diabetic participants per study enhanced the precision of individual prevalence estimates and reduced the influence of small studies with wide confidence intervals. The restriction to post-2015 publications ensured that the included studies reflect contemporary diagnostic practices and patient management contexts. Quality assessment using the NOS [5,6] and JBI Critical Appraisal Checklist [7] confirmed that the majority of the included studies were of moderate to high quality, supporting confidence in the reliability of reported estimates. The decision to stratify by diagnostic approach rather than pool across heterogeneous methods was a deliberate and appropriate analytical choice that accurately represents the data without obscuring clinically meaningful differences between subgroups.

Nonetheless, important limitations apply. The absence of a universal diagnostic standard across the included studies is the most significant constraint and directly precluded the quantitative meta-analysis of overall prevalence [28]. This is a recurrent challenge in DED research rather than a limitation specific to this review. Geographic skew toward Asia and Africa, with sparse representation from Europe, North America, and Australia. The restriction to English-language publications and the post-2015 date range introduce the possibility of publication bias, with relevant non-English or older studies potentially missed. Although subgroup pooled estimates were calculated, formal assessment of small-study effects using funnel plots or Egger's test was not conducted for any subgroup, given the high within-subgroup heterogeneity (I²) and substantial methodological diversity across diagnostic instruments and populations, which would render the visual interpretation of asymmetry unreliable regardless of study count. The potential for publication bias should be noted: studies reporting higher prevalence may be more likely to be published, which could inflate subgroup pooled estimates, particularly in the multimodal clinical test subgroup where study numbers are largest. The predominance of cross-sectional study designs limits any inference about causality, temporality, or disease progression. Finally, inconsistent reporting of key confounders including glycaemic control, DM duration, retinopathy severity, and insulin use prevented the deeper exploration of risk modifiers and precluded robust subgroup analyses. The 18 records not retrieved at the full-text stage represent a further limitation; however, as described in the Methods section, these were distributed across diagnostic categories and geographic regions and are unlikely to alter the direction of findings.

Implications of Findings

The high DED prevalence observed across studies, particularly those using objective clinical assessments, positions DED as an underrecognised and underdiagnosed complication of DM with direct consequences for vision-related quality of life. Current diabetes care pathways prioritise retinopathy screening, but the ocular surface is rarely assessed in a structured way. The evidence from this review supports integrating proactive, standardised DED screening into routine diabetes care, using accessible tools such as the OSDI questionnaire combined with TBUT or Schirmer's test. This aligns with the American Diabetes Association guidance on comprehensive ophthalmic evaluation that extends beyond retinal assessment alone.

Early identification of DED in patients with DM enables timely and targeted management. Interventions range from optimising glycaemic control to reduce neuropathic and inflammatory contributions to ocular surface therapies including artificial tears, lipid-based lubricants, anti-inflammatory agents such as topical cyclosporine and lifitegrast, and meibomian gland procedures where indicated [35]. Treating DED in this population preserves visual function, reduces ocular discomfort, and limits broader quality-of-life impairment in patients already carrying a high systemic disease burden.

In high-burden regions such as Asia and sub-Saharan Africa, targeted resource allocation toward ophthalmic screening within diabetic clinics offers a practical and high-yield public health intervention [36]. Achieving this requires multidisciplinary collaboration between endocrinologists, primary care providers, and ophthalmologists, with shared care pathways that normalise ocular surface assessment as part of standard diabetic review.

Future research should prioritise the adoption of standardised diagnostic criteria to generate meta-analysable prevalence data, longitudinal cohort designs to establish temporality and assess DED progression in relation to DM duration and glycaemic control, and consistent confounder reporting to enable subgroup and risk stratification analyses [28]. Expanded geographic representation, particularly from Europe, Latin America, and North America, would strengthen global applicability. Interventional trials evaluating the effect of early DED treatment on visual outcomes and quality of life in patients with DM are also warranted and currently absent from the literature.

## Conclusions

DED prevalence in patients with DM varies substantially by diagnostic method. Studies relying on ICD or administrative codes consistently underestimate prevalence relative to those using objective clinical tests or validated symptom questionnaires, reflecting differences in diagnostic sensitivity rather than true variation in disease burden.

DED is prevalent and clinically significant across the diverse populations represented in this review. Despite this, it remains underdiagnosed and underprioritised in routine diabetes care. The evidence supports integrating proactive, standardised DED screening into diabetes care pathways: early identification allows timely intervention, reduces ocular discomfort, preserves visual function, and limits quality-of-life impairment in a population already facing substantial disease burden.

## Appendices

Search strategies

Database: PubMed (MEDLINE)

Search date: June 2025

Search fields: Title/Abstract

Language restrictions: English (applied at screening stage)

Article type restrictions: None

Date range: 2015/01/01 to 2025/06/30

Concept 1: Dry Eye Disease ("dry eye"[Title/Abstract] OR "dry eye disease"[Title/Abstract] OR "dry eyes"[Title/Abstract] OR "keratoconjunctivitis sicca"[Title/Abstract] OR "ocular surface disease"[Title/Abstract] OR "tear film instability"[Title/Abstract] OR "dysfunctional tear syndrome"[Title/Abstract])

Concept 2: Diabetes Mellitus ("diabetes"[Title/Abstract] OR "diabetes mellitus"[Title/Abstract] OR "type 1 diabetes"[Title/Abstract] OR "type 2 diabetes"[Title/Abstract] OR "T1DM"[Title/Abstract] OR "T2DM"[Title/Abstract] OR "IDDM"[Title/Abstract] OR "NIDDM"[Title/Abstract])

Concept 3: Epidemiology and Frequency ("prevalence"[Title/Abstract] OR "epidemiology"[Title/Abstract] OR "incidence"[Title/Abstract] OR "frequency"[Title/Abstract] OR "occurrence"[Title/Abstract] OR "burden"[Title/Abstract])

Final Combined Search Strategy ("dry eye"[Title/Abstract] OR "dry eye disease"[Title/Abstract] OR "dry eyes"[Title/Abstract] OR "keratoconjunctivitis sicca"[Title/Abstract] OR "ocular surface disease"[Title/Abstract] OR "tear film instability"[Title/Abstract] OR "dysfunctional tear syndrome"[Title/Abstract]) AND ("diabetes"[Title/Abstract] OR "diabetes mellitus"[Title/Abstract] OR "type 1 diabetes"[Title/Abstract] OR "type 2 diabetes"[Title/Abstract] OR "T1DM"[Title/Abstract] OR "T2DM"[Title/Abstract] OR "IDDM"[Title/Abstract] OR "NIDDM"[Title/Abstract]) AND ("prevalence"[Title/Abstract] OR "epidemiology"[Title/Abstract] OR "incidence"[Title/Abstract] OR "frequency"[Title/Abstract] OR "occurrence"[Title/Abstract] OR "burden"[Title/Abstract]) AND ("2015/01/01"[PDAT] : "2025/06/30"[PDAT])

Database: Embase

Search date: June 2025

Search fields: Title/Abstract (ab,ti)

Subject Headings (Emtree, .sh)

Language restrictions: English (applied at screening stage)

Article type restrictions: None

Date range: 2015 to 2025

Concept 1: Dry Eye Disease ('dry eye'/exp OR 'keratoconjunctivitis sicca'/exp OR 'ocular surface disease'/exp OR dry NEAR/2 eye:ab,ti OR 'dry eye disease':ab,ti OR 'dry eyes':ab,ti OR 'keratoconjunctivitis sicca':ab,ti OR 'tear film instability':ab,ti OR 'dysfunctional tear syndrome':ab,ti)

Concept 2: Diabetes Mellitus ('diabetes mellitus'/exp OR 'insulin dependent diabetes mellitus'/exp OR 'non insulin dependent diabetes mellitus'/exp OR diabetes:ab,ti OR 'diabetes mellitus':ab,ti OR 'type 1 diabetes':ab,ti OR 'type 2 diabetes':ab,ti OR T1DM:ab,ti OR T2DM:ab,ti)

Concept 3: Epidemiology and Frequency ('prevalence'/exp OR 'epidemiology'/exp OR prevalence:ab,ti OR epidemiology:ab,ti OR incidence:ab,ti OR frequency:ab,ti OR occurrence:ab,ti)

Final Combined Search Strategy ('dry eye'/exp OR 'keratoconjunctivitis sicca'/exp OR 'ocular surface disease'/exp OR dry NEAR/2 eye:ab,ti OR 'dry eye disease':ab,ti OR 'dry eyes':ab,ti OR 'keratoconjunctivitis sicca':ab,ti OR 'tear film instability':ab,ti OR 'dysfunctional tear syndrome':ab,ti) AND ('diabetes mellitus'/exp OR 'insulin dependent diabetes mellitus'/exp OR 'non insulin dependent diabetes mellitus'/exp OR diabetes:ab,ti OR 'diabetes mellitus':ab,ti OR 'type 1 diabetes':ab,ti OR 'type 2 diabetes':ab,ti OR T1DM:ab,ti OR T2DM:ab,ti) AND ('prevalence'/exp OR 'epidemiology'/exp OR prevalence:ab,ti OR epidemiology:ab,ti OR incidence:ab,ti OR frequency:ab,ti OR occurrence:ab,ti) 

Database: Scopus

Search date: June 2025

Search fields: TITLE-ABS-KEY

Language restrictions: English (applied at screening stage)

Article type restrictions: None

Date range: 2015 to 2025

Concept 1: Dry Eye Disease TITLE-ABS-KEY("dry eye" OR "dry eye disease" OR "dry eyes" OR "keratoconjunctivitis sicca" OR "ocular surface disease" OR "tear film instability" OR "dysfunctional tear syndrome")

Concept 2: Diabetes Mellitus TITLE-ABS-KEY("diabetes" OR "diabetes mellitus" OR "type 1 diabetes" OR "type 2 diabetes" OR "T1DM" OR "T2DM" OR "IDDM" OR "NIDDM")

Concept 3: Epidemiology and Frequency TITLE-ABS-KEY("prevalence" OR "epidemiology" OR "incidence" OR "frequency" OR "occurrence" OR "burden")

Final Combined Search Strategy TITLE-ABS-KEY("dry eye" OR "dry eye disease" OR "dry eyes" OR "keratoconjunctivitis sicca" OR "ocular surface disease" OR "tear film instability" OR "dysfunctional tear syndrome") AND TITLE-ABS-KEY("diabetes" OR "diabetes mellitus" OR "type 1 diabetes" OR "type 2 diabetes" OR "T1DM" OR "T2DM" OR "IDDM" OR "NIDDM") AND TITLE-ABS-KEY("prevalence" OR "epidemiology" OR "incidence" OR "frequency" OR "occurrence" OR "burden") AND PUBYEAR > 2014 AND PUBYEAR < 2026

Database: Web of Science

Core collection search date: June 2025

Search fields: Topic (TS) - title, abstract, author keywords, Keywords Plus

Language restrictions: English (applied at screening stage)

Article type restrictions: None

Date range: 2015-01-01 to 2025-06-30

Concept 1: Dry Eye Disease TS=("dry eye" OR "dry eye disease" OR "dry eyes" OR "keratoconjunctivitis sicca" OR "ocular surface disease" OR "tear film instability" OR "dysfunctional tear syndrome")

Concept 2: Diabetes Mellitus TS=("diabetes" OR "diabetes mellitus" OR "type 1 diabetes" OR "type 2 diabetes" OR "T1DM" OR "T2DM")

Concept 3: Epidemiology and Frequency TS=("prevalence" OR "epidemiology" OR "incidence" OR "frequency" OR "occurrence")

Final Combined Search Strategy TS=("dry eye" OR "dry eye disease" OR "dry eyes" OR "keratoconjunctivitis sicca" OR "ocular surface disease" OR "tear film instability" OR "dysfunctional tear syndrome") AND TS=("diabetes" OR "diabetes mellitus" OR "type 1 diabetes" OR "type 2 diabetes" OR "T1DM" OR "T2DM") AND TS=("prevalence" OR "epidemiology" OR "incidence" OR "frequency" OR "occurrence") Timespan: 2015-01-01 to 2025-06-30
